# Optimization Control of the Color-Coating Production Process for Model Uncertainty

**DOI:** 10.1155/2016/9731823

**Published:** 2016-05-10

**Authors:** Dakuo He, Zhengsong Wang, Le Yang, Zhizhong Mao

**Affiliations:** ^1^College of Information Science and Engineering, Northeastern University, Shenyang, Liaoning 110004, China; ^2^State Key Laboratory of Synthetical Automation for Process Industries, Northeastern University, Shenyang 110004, China

## Abstract

Optimized control of the color-coating production process (CCPP) aims at reducing production costs and improving economic efficiency while meeting quality requirements. However, because optimization control of the CCPP is hampered by model uncertainty, a strategy that considers model uncertainty is proposed. Previous work has introduced a mechanistic model of CCPP based on process analysis to simulate the actual production process and generate process data. The partial least squares method is then applied to develop predictive models of film thickness and economic efficiency. To manage the model uncertainty, the robust optimization approach is introduced to improve the feasibility of the optimized solution. Iterative learning control is then utilized to further refine the model uncertainty. The constrained film thickness is transformed into one of the tracked targets to overcome the drawback that traditional iterative learning control cannot address constraints. The goal setting of economic efficiency is updated continuously according to the film thickness setting until this reaches its desired value. Finally, fuzzy parameter adjustment is adopted to ensure that the economic efficiency and film thickness converge rapidly to their optimized values under the constraint conditions. The effectiveness of the proposed optimization control strategy is validated by simulation results.

## 1. Introduction

Color-coated steel, also known as organic-coated steel, is widely applied in many fields, including the automotive industry, household appliances, and the real estate industry, because of its advantages of good anticorrosive properties and suitability for a wide range of environments. In the color-coating production process (CCPP), colored coatings are painted onto cleaned steel to form a thin film which imparts the desired properties to the steel.

Disadvantages of this production process, however, include high energy consumption, low efficiency, and wastage of raw materials. Process optimization, aimed at minimizing production costs and enhancing production efficiency, is therefore particularly important. The present work proposes an effective optimization control strategy to resolve actual CCPP problems to enhance the productivity and reduce production costs.

Optimization control of CCPP has rarely been reported in the literature, although a few researchers have investigated color-coating production in the iron and steel industry, known as color-coating production scheduling, which aims at maximizing productivity and minimizing production cost [1, 2].

Mechanistic models of the coating process should be developed for carrying out such optimization. Such process models play several significant roles: first, the modeling will analyze the mechanism of the coating process, identify the important operating variables and quality indexes of colored-coated steel, and analyze the relationship between them; second, such models can be regarded as data generators to produce the required production data used for simulation and analysis. As research objectives, the mechanistic models should be capable of linking the web film thickness—the key quality indicator—with the operating variables (the moving web speed, the roll speed, the tension, and the wrap angle). For a coating process operating in reverse mode with a web-to-roll speed ratio less than 1, the Reynolds lubrication equation predicts the film thickness on both the web and applicator roll [3]. This is an acknowledged color-coating process model [3–5] and is selected in this work to simulate the actual process. In addition, all production costs, including those of raw materials, electrical energy, and masking liquid, are considered in modeling the relationship between economic efficiency and the operating variables.

Production process data can be generated by mechanistic models. Process data analysis mainly aims at establishing predictive models that can be used to predict product indexes [6]. Partial least squares (PLS) regression has developed into a generalized multiple linear regression method, and it can be applied to establish the relationship between an input matrix *X* and an output matrix *Y* using a linear multivariate model [7–11]. PLS regression is a useful and effective regression technique in process modeling and product quality prediction because of its ability to analyze data with strongly collinear and noisy variables in both input *X* and output *Y* values [8]. In this work, the method of PLS regression was therefore used to develop predictive models of the coating process.

In this process optimization problem, economic efficiency is considered as the optimization objective, while the moving web speed, the roll speed, the tension, and the wrap angle are considered as decision variables, and the optimization model is established under the constraints of product quality. The method of sequential quadratic programming (SQP) [12, 13] was selected to solve this optimization problem. The optimal economic efficiency and the best set points, including the optimal film thickness and the corresponding operating variables, are obtained by solving the optimization problem.

The optimal economic efficiency is based on a PLS predictive model, and the actual optimal economic efficiency can be calculated by plugging the best set points into mechanistic models. There are some discrepancies between the forecast and actual values of the optimal economic efficiency obtained through optimization. In practice, however, one would be unlikely to develop an accurate (or even approximate) process model relating the input and output variables [14, 15], so predictive models are not capable of perfectly imitating the coating process. Although these models are established using actual production data, there is model uncertainty, which hampers process optimization. This can lead to the outcome that “optimal using the model” may not necessarily mean “optimal for the process” [16]. The robust optimization approach has been applied to address process optimization problems under conditions of uncertainty [17–23]. In this method, the uncertain parameters are represented using uncertainty sets [21, 22], and the rationale is to transform the process optimization problem with uncertain parameters into a deterministic optimization problem via discretizing the probability density function of the uncertain parameters [24, 25]. By introducing robust optimization, initial operating variables with better values of economic efficiency are obtained and the feasibility of the optimal solution is improved, which provides a preliminary solution to the model uncertainty problem.

Iterative learning control (ILC) is an effective technique for controlling systems of a repetitive nature with the requirement that a desired reference trajectory is precisely followed [26]. It has been widely utilized in industrial processes with repetitive motion because of its structural simplicity and effective learning ability [27–29]. Robust optimization, described above, partly overcomes the drawback of model uncertainty and improves economic performance. The results of robust optimization provide a better initial set point for ILC, which is then utilized to implement iterative optimization control of the CCPP to further improve economic efficiency by on-line adjustment of the operating variables. To overcome the drawback that traditional ILC is not capable of dealing with constraints, the film thickness is regarded as one of the tracking targets and the goal here is to deal with the quality constraint. The technique of iteratively tracking goal settings based on analysis of the predictive models is proposed. Fuzzy adaptive adjustment of target weighted parameters is implemented with the aim of rapidly converging the ILC and meeting the quality constraint. Using this approach, an effective optimization control strategy for CCPP is proposed to solve the problem of model uncertainty and improve economic efficiency by adjusting the operating variables.

## 2. Production Technology and Mechanistic Models of the Color-Coating Process

**Figure 1:**
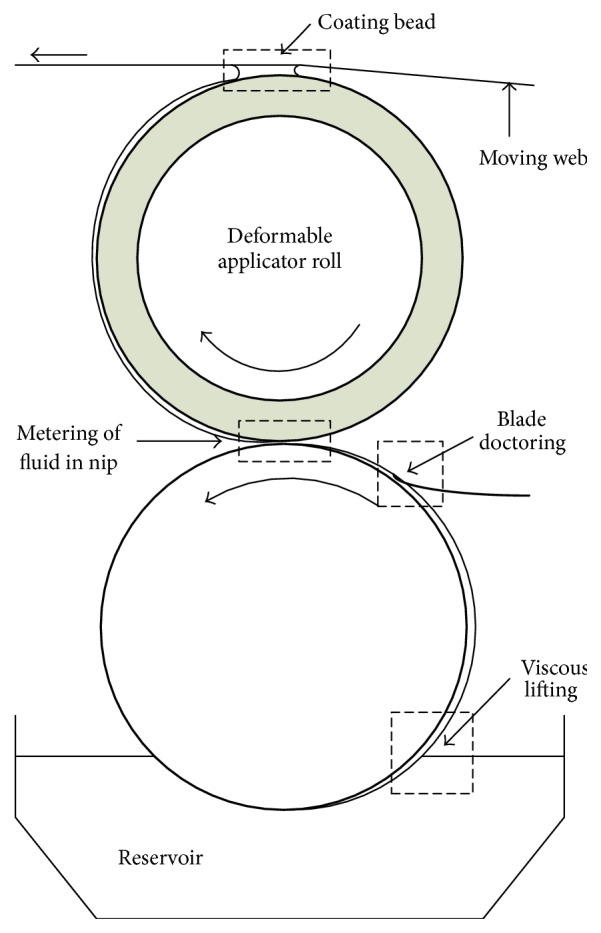
Schematic diagram of the color-coating process.

The kiss-roll coating system operating in reverse mode, named so because the web and the surface of applicator roll move in opposite directions, is the key technical equipment in color-coating production. It works on the principle of transferring the coating liquid film from the surface of an applicator roll onto a moving web [3, 5]. Because of its preferential stability characteristics, the reverse mode system is commonly used in industry [4]. A schematic diagram of a kiss-roll coating system operating in reverse mode is shown in Figure 1.

Film thickness is the significant quality indicator of CCPP. For the purpose of optimizing CCPP, the present work explores the impact of operating variables, such as the moving web speed, the roll speed, the tension, and the wrap angle, on the web film thickness.

**Figure 2:**
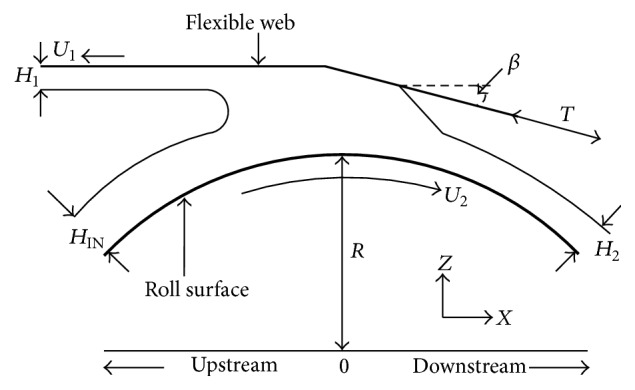
Schematic diagram of the flow domain of the coating process.

The flow domain of the coating process is shown schematically in Figure 2. Gaskell has developed a one-dimensional model [3] for a kiss-roll coating system operating in reverse mode, based on the following assumptions:

(1) The web is completely flexible and therefore free to bend when it is under pressure of the coating fluid.
(2) The wrap angle *β* is considered to be of a small positive number.
(3) The length scale in the direction of the *x*-axis is much greater than that in the direction of the *z*-axis.
(4) The coating fluid film that enters the bead is uniform and it cannot run back.
(5) The coating flow is steady and the effect of gravity is negligible.

The one-dimensional model derived to predict the film thickness on the web can be described as follows (the detailed derivation process is given in [3]):

(1) H1=RU2U1HINR−32μU1σ1−U1U22σTβ,U1U2<1,  β≠0,

where *H*_1_ is the film thickness on the web; *U*_1_, *U*_2_, *T*, and *β* are the operating variables, that is, the moving web speed, the roll speed, the tension, and the wrap angle, respectively; *R*, *H*_IN_, *μ*, and *σ* are the radius of the applicator roll, the film thickness of the coating bead entrance, the viscosity, and the surface tension of coating liquid, respectively, having the constant values shown in Table 1.

From the analysis of actual production, the mechanistic model of economic efficiency is described by the following form:

(2) $$\begin{array}{c} J=aF_{1}-bF_{2}-cF_{3}-dF_{4}, \end{array}$$

where *a*, *b*, *c*, and *d* are the prices of the finished color-coated steel product, the unprocessed steel, the coating, and industrial electricity, respectively; *F*_1_ is the yield of color-coated steel; and *F*_2_, *F*_3_, and *F*_4_ are the consumptions of unprocessed steel, the coating, and industrial electricity, respectively. *F*_1_, *F*_2_, *F*_3_, and *F*_4_ are denoted as follows in the model.

It is assumed that the weight of the steel plate is 40 kg per meter, so the yield of color-coated steel and the consumption of unprocessed steel per unit time are given by

(3) $$\begin{array}{c} F_{1}=F_{2}=40\times 3600\times U_{1}. \end{array}$$

The width of the steel is 1 m, so the consumption of the coating per unit time can be expressed as

(4) $$\begin{array}{c} F_{3}=3600\times H_{1}\times U_{1}. \end{array}$$

The power of the variable frequency motors used in color-coating production lines is 900 kW, and the power of the mains-frequency motors combined with that for lighting and other equipment is 300 kW. The consumption of industrial electricity per unit time is therefore

(5) $$\begin{array}{c} F_{4}=\frac{900U_{1}}{U_{1max}}+300, \end{array}$$

where *U*_1_ represents the speed of the moving steel and *U*_1max_ has a value of 3 m/s.

## 3. General Optimization of the Color-Coating Production Process

**Figure 3:**
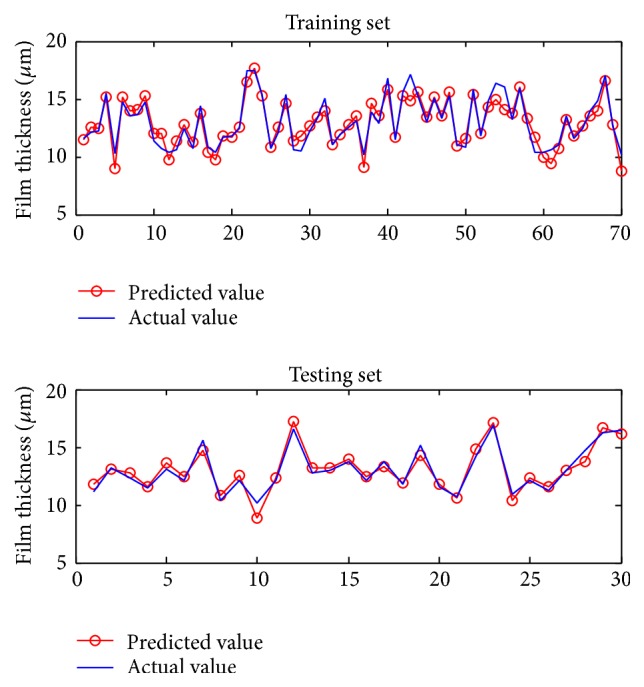
Simulation results of predictive model of film thickness based on PLS.

**Figure 4:**
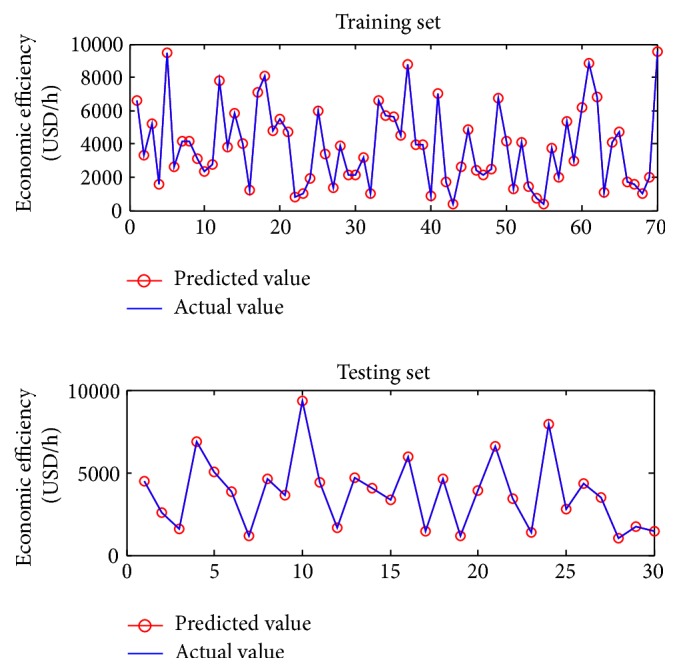
Simulation results of predictive model of economic efficiency based on PLS.

The PLS method is used to develop predictive models of the film thickness and economic efficiency. In the development of these predictive models, the input variables are the operating variables (the moving web speed, the roll speed, the tension, and the wrap angle) and the output variables are the film thickness on the web and the economic efficiency. Specifically, 70 samples were generated to train the PLS models and 30 samples to test them. The operating variables were first randomly generated within their constraints. The operating variables were plugged into the mechanistic models to calculate the corresponding film thickness and economic efficiency. The samples that contained operating variables and the corresponding film thickness or economic efficiency were used to establish the predictive models and test their accuracy. The model simulations are shown in Figures 3 and 4. The explicit form of the predictive models established can be expressed as

(6) JPLS1U1,U2,T,β=PLS1u=−12.42u1+5.92u2−0.0041u3+0.061u4+16.04,

(7) H1PLS2U1,U2,T,β=PLS2u=1.15u1−3.5u2+6.55u3−0.068u4−2.71,

where *u* = (*u*_1_, *u*_2_, *u*_3_, *u*_4_) = (*U*_1_, *U*_2_, *T*, *β*) and PLS_1_ and PLS_2_ are the PLS models of economic efficiency and film thickness, respectively.

The economic efficiency of production is treated as the optimization goal and the operating variables are treated as decision variables. Based on these predictive models of the coating process, the optimization model is derived as follows:

(8) max J=PLS1us.t. H1=PLS2u; H1min≤H1≤H1max; 0<u1u2<1; uimin≤ui≤uimax,i=1,2,3,4.

In this model, the web film thickness *H*_1_ is required to be within the range (*H*_1min_, *H*_1max_) and the operating variable *u* is regarded as *u*_min_ ≤ *u* ≤ *u*_max_. Combining all constraints, the ranges of the variables are listed in Table 2.

An SQP algorithm [12, 13] is adopted to solve the established optimization problem. The optimization results are shown in Table 3. However, owing to the inherent errors of the predictive model, it is difficult to achieve the desired economic efficiency by applying the optimized control trajectory obtained by this general optimization to the actual color-coating process. The actual values of the economic efficiency and quality indexes are shown in Table 4. The results in Tables 3 and 4 indicate that a discrepancy of 6.2% exists between the predicted and actual values of economic efficiency, which means that “optimal by the model” may not mean “optimal for the process,” as mentioned before.

## 4. Use of Robust Optimization to Solve the Problem of Model Uncertainty

To address the model uncertainty, the model error is regarded as the uncertain parameter and the optimization problem is described as the process optimization problem with the uncertain parameters. Optimization of the CCPP based on robust optimization is proposed. Specifically, the probability density function of the model error is obtained by making use of the modeling data, and the process optimization problem with the uncertain model error is solved by transforming it into a deterministic optimization problem by means of discretizing the probability density function of the model error. The model of robust optimization is expressed as

(9) max J=ϕ1u,d1s.t. H1=ϕ2u,d2; H1min≤H1≤H1max; 0<u1u2<1; uimin≤ui≤uimax,i=1,2,3,4,

where *d*_1_ and *d*_2_ are the uncertain parameter vectors of economic efficiency and film thickness, respectively and *ϕ*_1_ and *ϕ*_2_ represent the predictive models of economic efficiency and film thickness with uncertainty, respectively. The above robust optimization problem can be solved by discretizing the probability density functions *p*(*d*_1_) and *p*(*d*_2_). It is assumed that the number of points of the discretized probability density function is *D* and that *d*_*i*,*j*_ is the *j*th discrete value of the uncertain parameter that has a relative weight *ω*_*i*,*j*_  (∑*ω*_*i*,*j*_ = 1), where *i* = 1,2. This robust optimization problem can then be transformed into the following deterministic optimization problem:

(10) max J=PLS1u+∑j=1Dd1,jω1,js.t. H1=PLS2u+∑j=1Dd2,jω2,j; H1min≤H1≤H1max; 0<u1u2<1; uimin≤ui≤uimax,i=1,2,3,4.

**Figure 5:**
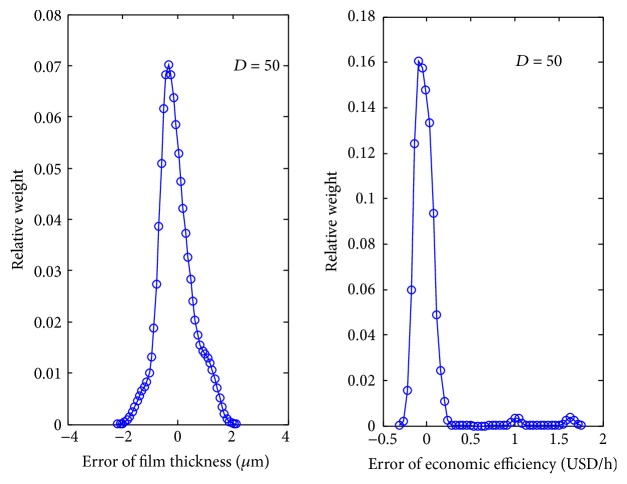
The probability density distribution of film thickness and economic efficiency.

The present work treats the model errors of web film thickness and economic efficiency as the uncertain parameters and analyzes their probability distributions. The probability density distributions *p*(*d*_1_) and *p*(*d*_2_) are obtained using the* ksdensity* function in* MATLAB*, as shown in Figure 5. The robust optimization results obtained using the SQP method are shown in Table 5. Applying the optimal operating variables based on robust optimization to a real CCPP, the actual values of quality indexes and economic efficiency are shown in Table 6. These results indicate that the actual economic efficiency based on the general optimization process is USD 8816, while the value given by robust optimization is USD 9189 (4.23% higher). Robust optimization therefore has the ability to limit the model uncertainty to a certain degree and enhance the feasibility of the optimal solution in the actual production process.

## 5. Use of Iterative Learning Control to Solve the Problem of Model Uncertainty

Although robust optimization improves the feasibility of the solution, some model uncertainty still exists: it is unknown whether the robust optimization results represent optimal solutions to this problem. It is therefore likely that the economic efficiency can be improved further. To further eliminate the impact of the model uncertainty, the iterative nature of numerical optimization and the repetitive properties of the coating process can be utilized. ILC is introduced to improve the economic efficiency.

### 5.1. Principle of Iterative Learning Control

In terms of the relationship between the input and output variables obtained from the PLS, the system output *y*_*k*_^*T*^ of the *k*th trial can be expressed as

(11) $$\begin{array}{c} y_{k}^{T}=u_{k}^{T}\theta +{\hat{e}}_{k}^{T}={\hat{y}}_{k}^{T}+{\hat{e}}_{k}^{T}, \end{array}$$

where *u*_*k*_^*T*^ is the input control trajectory of the *k*th trial; ${\hat{y}}_{k}^{T}$ is the predictive value of system output; *k* is the number of iterations; *θ* is the linear regression matrix coefficient between the input and output control trajectories obtained from the PLS algorithm; and ${\hat{e}}_{k}^{T}$ is the predictive model error of the *k*th trial.

Considering the influence of the established model on the system, the purpose of ILC is to reduce the impact of model uncertainty by handling the operating variables between the trials and ensuring that the system output follows the desired value. To enable the system output to attain the desired value as early as possible and obtain the preferred input control trajectory precisely and stably, we solve the following quadratic form, based on the predictive model, to renew the input control trajectory from the *k*th trial to the (*k* + 1)th trial:

(12) min⁡Δuk+1 Δe^k+1TQ2+ΔUkR2,

where *Q* = *q* × *I*_*n*_, *R* = *r* × *I*_*n*_ (*r* and *q* are positive scalars, and *I*_*n*_ is an *n*-dimensional positive definite diagonal matrix), and ${\hat{e}}_{k+1}^{T}=y_{d}^{T}-{\hat{y}}_{k+1}^{T}=y_{d}^{T}-u_{k+1}^{T}\theta$, and *y*_*d*_^*T*^ is the desired system output trajectory. This can be determined by taking the derivative with respect to (12):

(13) $$\begin{array}{c} \Delta u_{k+1}^{T}={\hat{e}}_{k}^{T}Q\theta {\left(\;\theta^{T}Q\theta +R\;\right)}^{-1}. \end{array}$$

Using (13), the increment Δ*u*_*k*+1_^*T*^ is calculated from the output prediction error ${\hat{e}}_{k}^{T}$ of the *k*th trial. Because an error exists between the established predictive model and the actual process, it is necessary to amend this value using the actual error *e*_*k*_^*T*^ of the system output. The auxiliary variable trajectory of the (*k* + 1)th trial can be revised to be

(14) $$\begin{array}{c} \Delta u_{k+1}^{T}=\lambda_{k}e_{k}^{T}Q\theta {\left(\;\theta^{T}Q\theta +R\;\right)}^{-1}, \end{array}$$

and then

(15) $$\begin{array}{c} u_{k+1}^{T}=u_{k}^{T}+\lambda_{k}e_{k}^{T}Q\theta {\left(\;\theta^{T}Q\theta +R\;\right)}^{-1}, \end{array}$$

where *λ*_*k*_ is the weight factor. The rate of convergence can be adjusted by changing the value of *λ*_*k*_. Equation (14) gives the iterative calculation of the increment Δ*u*_*k*+1_^*T*^, which guarantees that the error of system output *e*_*k*_^*T*^ converges gradually.

### 5.2. Iterative Learning Control Applied to the Color-Coating Production Process

In the actual production process, certain requirements of the quality indexes keep the film thickness within strict boundaries; however, the basic algorithm of traditional ILC does not have the ability to solve optimization problems with constraints. To overcome this problem, we attempted to analyze the relationship between the predictive models of film thickness and economic efficiency and determine the special relationship between them. Combining (6) and (7), the economic efficiency *J* can be denoted by *H*_1_, *u*_2_, *u*_3_, and *u*_4_:

(16) JfH1,u2,u3,u4=−927.52H1+1986.55u2−3.84u3+56.94u4+14608.87.

From (16), it can be seen that

(17) $$\begin{array}{c} \frac{\partial f}{\partial H_{1}}=-927.52<0, \end{array}$$

so the value of economic efficiency is inversely proportional to that of the film thickness. In addition, from (6) and (7), it can be concluded that

(18) ∂PLS1∂u1∂PLS2∂u1<0,∂PLS1∂u2∂PLS2∂u2<0,∂PLS1∂u3∂PLS2∂u3<0,∂PLS1∂u4∂PLS2∂u4<0.

According to (17) and (18), under conditions of keeping the operating variables *u*_2_, *u*_3_, and *u*_4_ constant, the economic efficiency will increase with decreasing film thickness. In addition, the effects of the operating variables on film thickness and economic efficiency show opposite trends. Therefore, if we want to achieve optimal economic efficiency, the film thickness should be as thin as possible. This conclusion is not only derived from this theoretical analysis, but also verified through production experience. There is a constraint for the film thickness in actual production—it cannot be infinitely decreased; the optimized economic efficiency should therefore correspond to the minimum practical value of film thickness. However, this optimal economic efficiency cannot be obtained by directly tracking the minimum value of film thickness because there are multiple sets of operating variables corresponding to this minimum value; that is, the minimum value of film thickness can correspond to multiple values of economic efficiency by adjusting the operating variables. The economic efficiency obtained by directly tracking the minimum value of film thickness may therefore not provide the optimal solution.

Given the specific relationship between economic efficiency and film thickness, the present work proposes a method of transforming this optimal control problem with quality constraints into a double objective optimization control problem. In the ILC process, the two objectives (economic efficiency and film thickness) are traced simultaneously; the drawback that ILC cannot solve an optimization problem with constraints is overcome by treating the film thickness as one of the tracking targets. The system outputs, therefore, consist of film thickness *H*_1_ and economic efficiency *J*. The double objective optimization problem can be described by the following form:

(19) min⁡ q1Jd−J2+q2H1min−H12+ΔUR,

where *q*_1_ and *q*_2_ are the weights of economic efficiency and film thickness in matrix *Q*, respectively; *J*_*d*_ is the desired value of economic efficiency; and *H*_1min_ is the minimum film thickness.

Using the above analysis, the tracking target of film thickness is set to the minimum value (*H*_1*d*_ = *H*_1min_ = 10 *μ*m); but setting the tracking target of economic efficiency is difficult. Exceeding economic efficiency settings will lead to the film thickness exceeding the restricted range while lower settings will reduce the economic efficiency of actual production, so the tracking target of economic efficiency cannot be set optionally. The actual value of film thickness based on the optimal operating variables is greater than 10 *μ*m, whether determined by robust or general optimization, as shown in Tables 4 and 6. Under the current operating variables, the economic efficiency is first calculated with an assigned film thickness of 10 *μ*m. Thereafter, the increment of economic efficiency is calculated using the following equation:

(20) $$\begin{array}{c} \Delta J=f\left(\;H_{1act},u_{2},u_{3},u_{4}\;\right)-f\left(\;H_{1min},u_{2},u_{3},u_{4}\;\right), \end{array}$$

where *H*_1act_ is the actual film thickness based on the optimized results; *H*_1min_ is the minimum value of film thickness, and *H*_1min_ = 10 *μ*m, which gives Δ*H*_1_ = *H*_1act_ − *H*_1min_.

The tracking target of economic efficiency *J*_*d*_ can therefore be set as

(21) $$\begin{array}{c} J_{d}=J_{dp}+\Delta J, \end{array}$$

where *J*_*dp*_ is the previous desired economic efficiency.

With this determination of tracking target of economic efficiency, ILC can be used to simultaneously trace the targets of economic efficiency and film thickness. When the actual economic efficiency approaches the target value *J*_*d*_  (*J*_*d*_ − *J* < *ε*_1_), attention should be given to the convergence of the film thickness and the value of Δ*H*_1_. Here *ε*_1_ has a small positive value, a factor that decides whether to carry out the next decision regarding the renewal of the tracking target of economic efficiency. In this study, a threshold level *ε*_2_ is used as the factor to decide whether to sequentially increase the tracking target of economic efficiency. If the value of Δ*H*_1_ is greater than *ε*_2_ when the actual economic efficiency approaches the desired value *J*_*d*_, that is, the condition *J*_*d*_ − *J* < *ε*_1_ is met, then the tracking target of economic efficiency is increased once again and is updated using

(22) ΔJl+1=fH1act,l,u2,l,u3,l,u4,l−fH1min,u2,l,u3,l,u4,l,Jd,l+1=Jd,l+ΔJl+1,

where *l* is the renewal number for the economic efficiency (*l* = 0,1, 2,3,…).

ILC is then applied to improve the economic efficiency of actual production process. The optimization problem is expressed as

(23) min⁡ q1Jd,l−Jl2+q2H1min−H1,l2+ΔUlR.

In this way, the repetitive ILC process will always continue until the value of Δ*H*_1_ is less than or equal to *ε*_2_. One can therefore see that the present work takes the approach of enhancing the tracking target settings of economic efficiency or updating its desired values several times until the actual film thickness is close to its minimum value.

After determining how to set the target of economic efficiency, the next thing to consider is how to precisely and rapidly converge the economic efficiency and film thickness to the set goals. As is known, the convergence rate can be changed arbitrarily by varying the value of weight *Q* [30]: a larger value will increase the rate of convergence. It is assumed that the weight of economic efficiency is *q*_1_  (0 ≤ *q*_1_ ≤ *α*) and the weight of the film thickness is *q*_2_ = 1 − *q*_1_. The convergence rates of economic efficiency can be changed by adjusting the weight *q*_1_. The convergence rate of economic efficiency will increase as weight *q*_1_ increases, but if *q*_1_ is set as an excessive constant, the control of film thickness will be ignored, which results in film thickness being out of its restrained range. In such cases, if the film thickness is kept within its restrained range, then the economic efficiency will be lower. On the contrary, if *q*_1_ is too small, then economic efficiency will be ignored in the ILC process compared with film thickness and its convergence rate will be too slow. This implies a lower economic efficiency in actual production. The value of *q*_1_ therefore changes constantly in the ILC process. When the tracking errors of the economic efficiency and film thickness are high, the value of the weight should also be high. Moreover, the value of the weight should decrease as the tracking error of film thickness decreases to ensure that the film thickness is kept within the range of its constraints.

**Figure 6:**
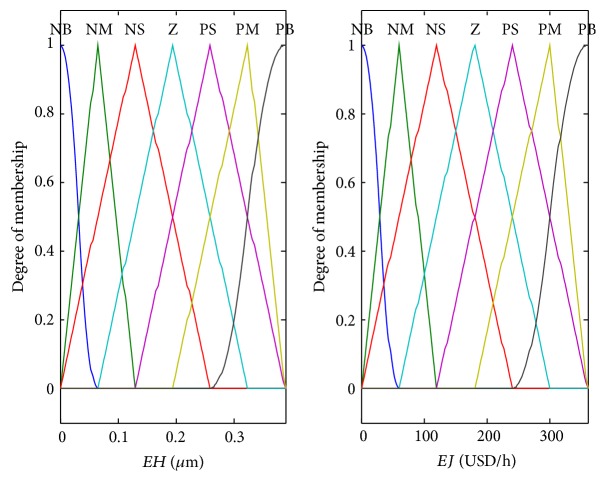
Membership functions of the errors.

**Figure 7:**
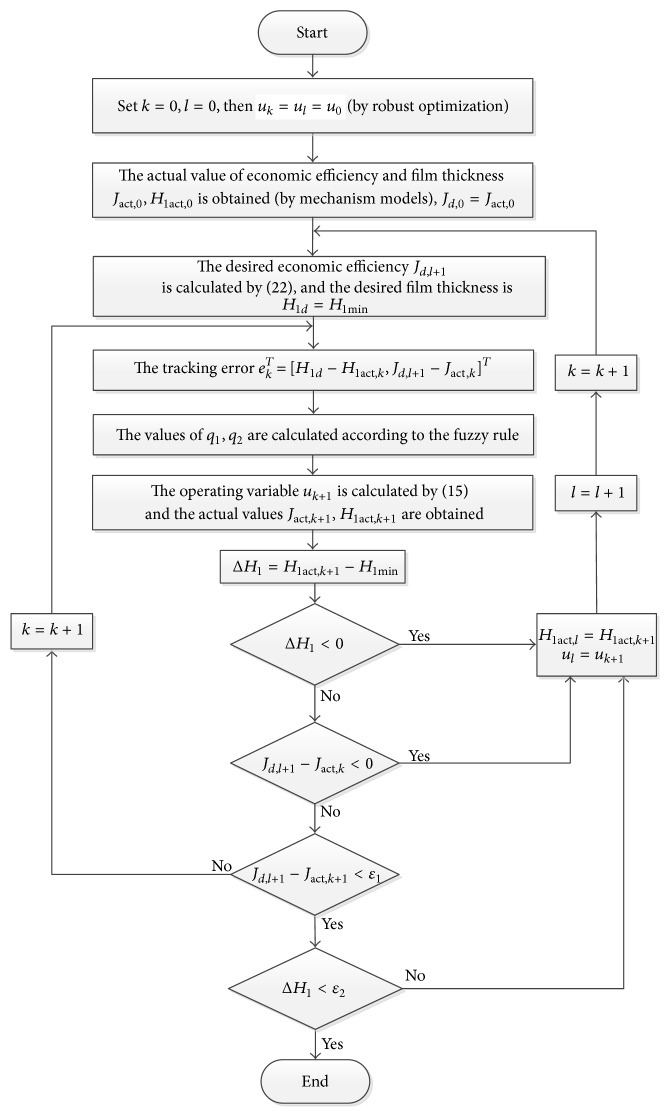
Program flow diagram of iterative learning control.

Based on this principle, the idea of fuzzy adjustment is introduced to adjust the weight *q*_1_, which enables economic efficiency and film thickness to converge rapidly to their desired values. The fuzzy adjustment rules are shown in Table 7, where* EH* and* EJ* represent the absolute values of the tracking errors of film thickness and economic efficiency, respectively. The membership functions of the absolute error values of film thickness and efficiency in the first stage of the ILC process are shown in Figure 6. In brief, the program flow diagram of the proposed ILC method to overcome this model uncertainty is shown in Figure 7.

**Figure 8:**
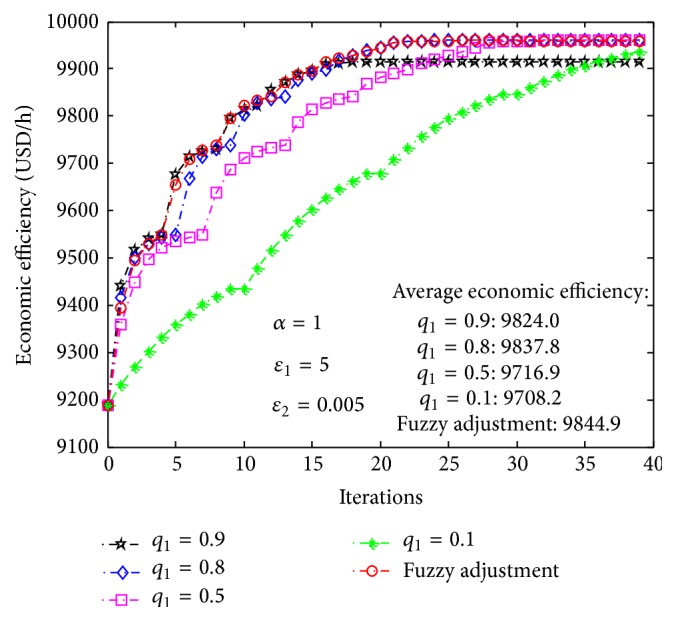
Evolution of economic efficiency based on iterative learning control.

**Figure 9:**
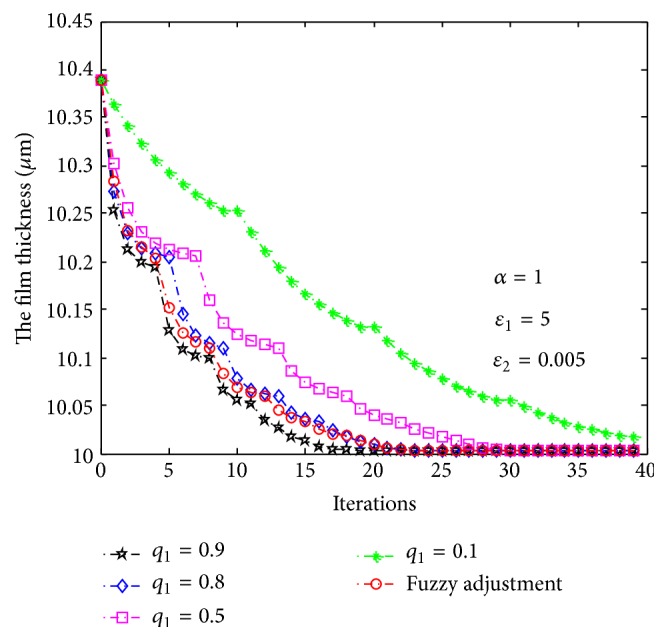
Evolution of film thickness based on iterative learning control.

Figures 8 and 9, respectively, show the evolutions of economic efficiency and film thickness from batch to batch based on ILC. The red curves in the figures describe their evolutions based on fuzzy parameter adjustment and the other curves show the trajectories of evolutions when *q*_1_ is set at various fixed values. These simulation curves indicate that the rate of convergence of economic efficiency increases as *q*_1_ increases, but if the value of *q*_1_ is too large (*q*_1_ = 0.9, e.g.), the convergence rate will be so fast that the film thickness will exceed the restrained range or the economic efficiency will be lower when the constraint of film thickness is satisfied; conversely, if the value of *q*_1_ is too small (*q*_1_ = 0.1), the convergence rate will be too slow, which leads to lower economic efficiency. The idea of fuzzy adjustment is therefore introduced to adjust the value of *q*_1_ and then improve the value and convergence rate of economic efficiency (shown as the red curves in Figures 8 and 9).

The initial values of economic efficiency and film thickness are USD 9189 and 10.39 *μ*m, respectively. Based on the proposed ILC method, the first tracking target of economic efficiency is USD 9550. After about five batches, the economic efficiency of actual production achieved the first tracking target, and accordingly the film thickness was reduced to 10.2 *μ*m. In the same way, after updating the tracking target of economic efficiency several times, the economic efficiency converges to its optimal value of USD 9960, which corresponds to a film thickness of 10 *μ*m. According to the average economic efficiency of all batches, when *q*_1_ is set as a constant, the economic efficiency is lower than that based on fuzzy parameter adjustment: this verifies the superiority of fuzzy adjustment parameters.

**Figure 10:**
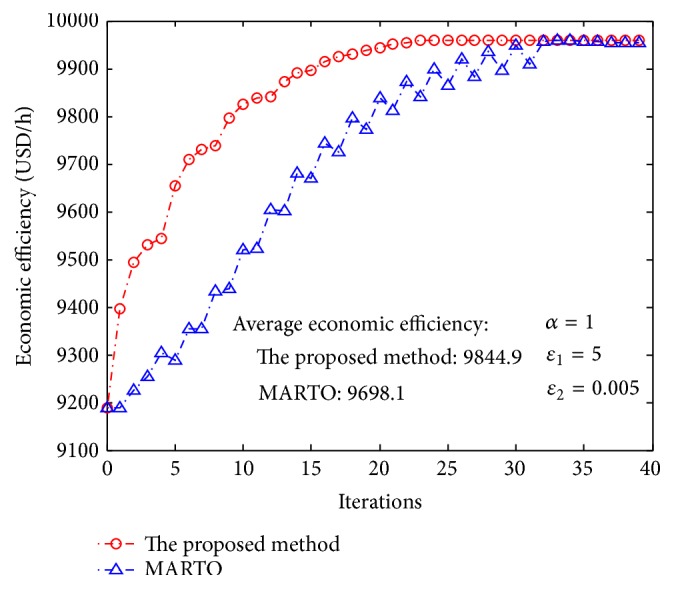
Comparison of economic efficiency between the proposed method and MARTO.

**Figure 11:**
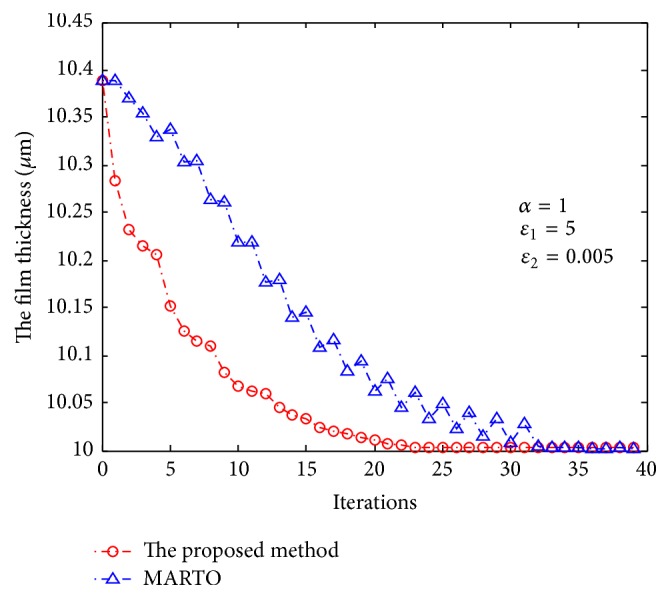
Comparison of film thickness between the proposed method and MARTO.

In addition to ILC, modifier adaptation real-time optimization (MARTO) [31, 32] is another optimization control method to address model uncertainty and a typical adaptive optimization approach [33]. The principle of modifier adaptation is to modify the objective and constraint functions between successive optimization iterations using the so-called modifiers representing the difference between the actual plant values and the predicted values in order to generate set points converging to the true optimum of the plant [31, 34]. However, MARTO has not been applied to the optimization control of CCPP. In order to test the performance of the proposed method in this paper, this method is implemented and applied to the optimization control of CCPP. The results have been compared with that of the proposed method (Figures 10 and 11). The simulations reveal that the method proposed in this paper was superior to MARTO in handling the model uncertainty of CCPP.

## 6. Conclusions

In this paper, an effective optimization control strategy for the CCPP is proposed. First, a mechanistic model of CCPP is introduced to simulate the actual production process and produce the process data. Predictive models of film thickness and economic efficiency are then developed using a PLS method. To manage the model uncertainty, the robust optimization approach is introduced to obtain a preferred initial solution and enhance the feasibility of the optimized solution. The effectiveness of robust optimization is validated by simulation results. To further refine the solution to the model uncertainty, ILC is applied. The film thickness, as the quality indicator, is constrained in the CCPP. Because the traditional ILC approach is not capable of dealing with constraints, the constrained film thickness is transformed into one of the tracking targets, defined such that optimal economic efficiency is achieved when the film thickness reaches its minimum value of 10 *μ*m. The desired film thickness is set at 10 *μ*m, and the goal setting of economic efficiency is updated continuously based on the desired film thickness until this reaches the desired value. Finally, the use of fuzzy parameter adjustment is adopted, by which the economic efficiency and film thickness are rapidly converged to their optimal values under the constraint conditions. The simulation results indicate that the proposed optimization control strategy can effectively solve the model uncertainty problem in CCPP. The proposed method provides a strong theoretical basis for optimizing actual production parameters.

The proposed optimization control strategy is easy to be implemented in practical engineering applications because it requires less accuracy of the model. Parameter *λ*_*k*_ should be paid more attention, since the setting of its initial value affects the performance of the proposed method. An excessive value of *λ*_*k*_ may produce unqualified products at the beginning of iterative learning control, while a rather small value may generate a lower economic efficiency. Therefore, the initial *λ*_*k*_ should be set according to the actual production situation. Similarly, the fuzzy rules should also be made based on the actual situation. Although this optimization control strategy is proposed for CCPP, it can also be applied to the process optimization problems, which possess one quality constraint.

## Figures and Tables

**Table 1:** Values of the model parameters.

Model parameters	*R* (m)	*H*_IN_ (*µ*m)	*µ* (mPa·s)	σ (N/m)
Value	0.1	6	0.998	0.033

**Table 2:** Ranges of the variables.

Quality constraint	Operating variables			
*H*_1_ (*µ*m)	*u*_1_ (m/s)	*u*_2_ (m/s)	*u*_3_ (N)	*u*_4_ (°)
[10, 17.5]	[0.3, 3]	[0.3, 3]	[720, 840]	[0, 5]

**Table 3:** Results of general optimization.

Operating variables				Quality index	Economic efficiency
*u*_1_ (m/s)	*u*_2_ (m/s)	*u*_3_ (N)	*u*_4_ (°)	*H*_1_ (*µ*m)	*J* (USD)
1.70	2.99	720	5	10	9398.8

**Table 4:** Actual values of the quality index and economic efficiency based on general optimization.

Operating variables				Quality index	Economic efficiency
*u*_1_ (m/s)	*u*_2_ (m/s)	*u*_3_ (N)	*u*_4_ (°)	*H*_1_ (*µ*m)	*J* (USD)
1.70	2.99	720	5	10.59	8816

**Table 5:** Results of robust optimization.

Operating variables				Quality index	Economic efficiency
*u*_1_ (m/s)	*u*_2_ (m/s)	*u*_3_ (N)	*u*_4_ (°)	*H*_1_ (*µ*m)	*J* (USD)
1.73	2.99	720	5	10	9582.4

**Table 6:** Actual values of the quality index and economic efficiency based on robust optimization.

Operating variables				Quality index	Economic efficiency
*u*_1_ (m/s)	*u*_2_ (m/s)	*u*_3_ (N)	*u*_4_ (°)	*H*_1_ (*µ*m)	*J* (USD)
1.73	2.99	720	5	10.39	9189

**Table 7:** Fuzzy control rules.

*q*_1_	*EJ*						
*EH*	NB	NM	NS	Z	PS	PM	PB
NB	NB	NB	NB	NB	NB	NB	NB
NM	PS	PS	PS	PM	PB	PB	PB
NS	PS	PS	PM	PM	PB	PB	PB
Z	PM	PM	PM	PM	PB	PB	PB
PS	PM	PM	PM	PM	PB	PB	PB
PM	PB	PB	PB	PB	PB	PB	PB
PB	PS	PS	PS	PS	PS	PS	PS
